# Dietary Patterns by Level of Maternal Education and Their Contribution to BMI, Fat Mass Index, and Fat-Free Mass Index at Age 5 and the Longitudinal Association with BMI at Age 10

**DOI:** 10.3390/nu16193242

**Published:** 2024-09-25

**Authors:** Viyan Rashid, Mary Nicolaou, Arnoud P. Verhoeff, Peter J. M. Weijs, Martinette T. Streppel

**Affiliations:** 1 Faculty of Sports and Nutrition, Center of Expertise Urban Vitality, Amsterdam University of Applied Sciences, Dr. Meurerlaan 8, 1067 SM Amsterdam, The Netherlands; p.j.m.weijs@hva.nl (P.J.M.W.); m.t.streppel@hva.nl (M.T.S.); 2 Department of Public and Occupational Health, Amsterdam University Medical Centers, University of Amsterdam, Van der Boechorststraat 7, 1081 BT Amsterdam, The Netherlands; m.nicolaou@amc.uva.nl; 3 Sarphati Amsterdam, Department of Healthy Living, Public Health Service (GGD) Amsterdam, Nieuwe Achtergracht 100, 1018 WT Amsterdam, The Netherlands; averhoeff@ggd.amsterdam.nl; 4 Department of Sociology, University of Amsterdam, Nieuwe Achtergracht 166, 1018 WV Amsterdam, The Netherlands; 5 Department Nutrition & Dietetics, Amsterdam University Medical Centers, Location VUmc, Amsterdam Public Health Research Institute, De Boelelaan 1117, 1081 HV Amsterdam, The Netherlands

**Keywords:** dietary patterns, children, pre-school children, maternal education, SES, socio-economic status, BMI, overweight, FMI, FFMI

## Abstract

Background: Our aim was to identify dietary patterns by the level of maternal education that contribute to BMI, fat mass index (FMI), and fat-free mass index (FFMI) in children at age 5 and to assess if these dietary patterns are related to BMI at age 10. Methods: Per group (low/middle/high level), Reduced Rank Regression (RRR) was used to derive dietary patterns for the response variables BMI z-score, FMI, and FFMI in 1728 children at age 5 in the Amsterdam Born Children and their Development (ABCD) cohort. Regression analyses were then used to determine the association with BMI at age 10. Results: In each group, pattern 1 was characterized by its own cluster of food groups. Low: water/tea, savory snacks, sugar, low-fat meat, and fruits; middle: water/tea, low-fat cheese, fish, low-fat dairy, fruit drink, low-fat meat, and eggs; and high: low-fat cheese, fruits, whole-grain breakfast products, and low-fat and processed meat. Additionally, in each group, pattern 1 was positively associated with BMI z-scores at age 10 (low: β ≤ 0.43 [95% CI ≤ 0.21; 0.66], *p* < 0.001, middle: β ≤ 0.23 [0.09; 0.36], *p* ≤ 0.001, and high: β ≤ 0.24 [0.18; 0.30], *p* < 0.001). Conclusions: The dietary patterns stratified by the level of maternal education are characterized by different food groups. But in all the groups, pattern 1 is positively associated with BMI at age 10.

## 1. Introduction

Differences in BMI and body composition in children are unequally distributed across socio-economic status (SES) groups and these differences already start at an early age [1,2]. Underweight as well as overweight and obesity are risk factors for the development of diseases [3,4]. Dietary patterns are important determinants of BMI and body composition in childhood [5], and they are associated with SES [6]. In several birth cohort studies, a higher consumption of a healthy dietary pattern in young children was associated with a higher SES, while a higher consumption of an unhealthier/junk pattern was associated with a lower SES [7,8]. We observed similar findings in the Amsterdam Born Children and their Development (ABCD) cohort, where data on dietary intake was available at age 5 [9]. 

A number of studies have observed cross-sectional or longitudinal associations between dietary patterns and BMI and body composition in children from Western European countries [10,11,12,13,14]. Surprisingly, some studies have observed that the consumption of ‘healthy’ dietary patterns increased levels of overweight or obesity [10,11]. Also, the consumption of dietary patterns high in processed foods decreased the prevalence of overweight or obesity [10]. We also observed this tendency in the ABCD cohort [13]. When children were 5 years old, higher scores on a healthy dietary pattern were associated with increased weight development, and higher scores on a full-fat dietary pattern were associated with decreased weight development between the ages of 5 and 10 years [13]. Crucially, these described dietary patterns were obtained via an a posteriori method (PCA) or an a priori method (diet score). To understand which dietary components are most associated with BMI and body composition in children, we applied Reduced Rank Regression (RRR) analysis. RRR combines the a posteriori and a priori methods. Dietary patterns are identified in an exploratory way but are based on a priori knowledge in the form of pre-selected response variables that are thought to link dietary patterns to disease risk [15]. Few studies have derived dietary patterns using RRR that were related to BMI and body composition. However, these RRR-derived dietary patterns were obtained in a different age category [16] or nutrient intakes were used as response variables [17]. 

Deriving dietary patterns that explain most of the variation in BMI, fat mass index (FMI), and fat-free mass index (FFMI) cross-sectionally will enable us to identify high-risk dietary patterns that are associated with BMI later in life (longitudinally). In addition, deriving dietary patterns stratified by the level of maternal education as a proxy for SES will provide us with information about differences in food items that contribute to the explained variation in BMI and measures of body composition across the different groups. This information might give health care professionals starting points for targeted interventions. 

Therefore, it is of interest to (1) identify dietary patterns stratified by the level of maternal education which explain most variance in BMI, FMI, and FFMI at age 5, and (2) to assess to which extent these dietary patterns are related to BMI and weight status at age 10. 

## 2. Materials and Methods

### 2.1. Study Design and Population

**Figure 1:**
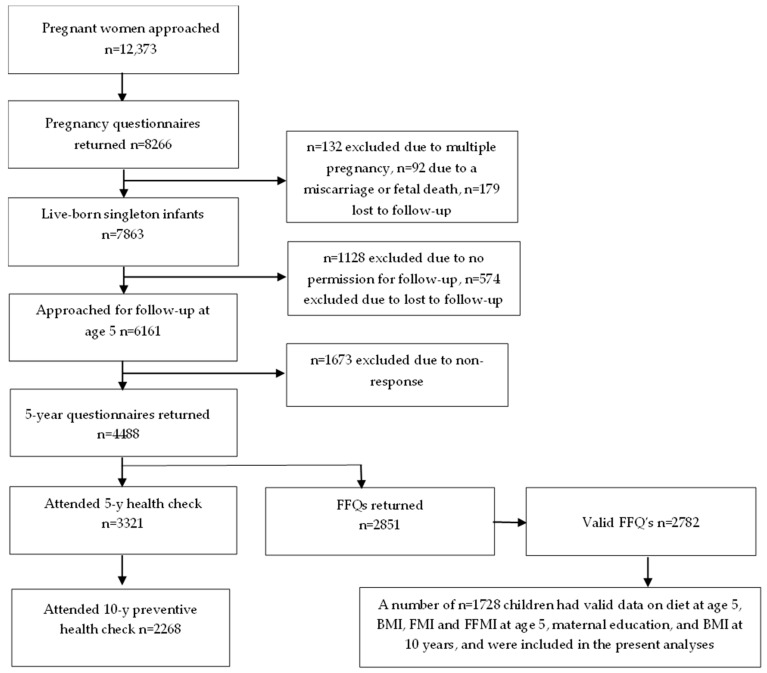
Flow chart of the participants included in the present analyses (n ≤ 1728).

This study was part of the ABCD cohort, a large ongoing community-based birth cohort (http://www.abcd-study.nl/ (accessed on 3 August 2024)). The design of the cohort study has been described previously [18]. Between January 2003 and March 2004, all pregnant women who were living in Amsterdam were invited to participate in the ABCD cohort at their first parental care visit. Of the 12,373 women that were approached, a number of 8266 women filled out a ‘pregnancy questionnaire’. At age 5, the addresses of 6161 mothers were retrieved from the Youth Health Care registry. A ‘5-year questionnaire’ was sent to the woman’s home address and a number of 4488 questionnaires were filled out by the mothers. These woman’s received an invitation for a ‘health check’ which was attended by 3321 children and a self-administered Food Frequency Questionnaire (FFQ). A number of 2851 mothers completed the FFQ. At age 10, the parents received an invitation for a preventive health check of their child, arranged by Youth Health Care part of the Public Health Service Amsterdam, carried out at their primary school. A number of 1728 children had valid data on diet at age 5; BMI, FMI, and FFMI at age 5; maternal education; and BMI at 10 years, and were included in the present analyses (Figure 1). 

### 2.2. Assessment of Dietary Intake and Patterns 

When the children were 5 years old, their mother completed a 71-item FFQ that was developed and validated by TNO Food (Zeist, The Netherlands) [19]. Per food item, the consumption frequency, type of product, and portion size consumed during the previous 4 weeks were reported. The given frequency options were ‘never’, ‘less than once a week’, ‘once a week’, ‘2–3 times a week’, ‘4–5 times a week’, and ‘6–7 times a week’. Based on the data clearance protocol developed by TNO Food, the returned FFQs were scanned. Data on the amounts (g/day) of products consumed and energy intake was calculated using the Dutch Food Composition Database (NEVO) 2010 [20]. A number of 41 food groups were composed and per food group, the energy-adjusted intake (g/d) was calculated [9].

Reduced Rank Regression (RRR) analyses identify dietary patterns in an exploratory way, but use pre-selected response variables that are thought to link dietary patterns to disease risk [15]. For the first research objective, RRR was used to identify dietary patterns for the response variables BMI z-score, FMI, and FFMI at age 5. This analysis was conducted separately per level of maternal education. Initially, three dietary patterns were derived that explained the maximal variance in the response variables. As the third RRR-derived dietary pattern contributed <10% to the total variance explained, it was left out of the subsequent analyses. RRR analyses produce a continuous score for each dietary pattern per individual, which represents the extent to which the dietary intake adheres to the dietary pattern. 

### 2.3. Assessment of BMI

When the children were 5 years old, height and weight were measured during the ABCD ‘health check’. The children were measured by trained researchers according to standard protocols using a Leicester portable height measurer (Seca, Kettering, UK) and a Marsden weighing scale (model MS-4102, Advanced Health Products, London, UK). When the children were 10 years old, height and weight were measured by a health professional during the preventive health check at the children’s primary school. During all the measures, the children wore light clothing. 

Height and weight were calculated into BMI scores [weight (kg)/height (m)^2^]. To categorize BMI scores into underweight, normal weight, and overweight/obesity, age- and sex-specific BMI cut-offs from the International Obesity Task Force [3,21] were used. Also, the BMI scores were converted to age- and sex-specific BMI z-scores (SD scores) by comparison with the ‘World Health Organization standards’ [22]. 

### 2.4. Assessment of FMI and FFMI

When the children were 5 years old, components of body composition were measured to calculate FMI and FFMI during the ABCD ‘health check’ where children were measured by trained researchers according to standard protocols. Fat mass (FM) with arm-to-leg bioelectrical impedance analysis (BIA) was measured (Bodystat 1500 MDD machine (Bodystat Inc., Douglas, UK)), fat-free mass (FFM) was measured (weight (kg)—FM), and fat mass index (FMI) (fat mass (kg)/height (m)^2^) and fat-free mass index (FFMI) (fat-free mass (kg)/height (m)^2^) were calculated. 

### 2.5. Assessment of Socio-Economic Status

The level of maternal education was obtained via the ‘5-year questionnaire’ and was used as a proxy for SES. It was defined as the number of years of post-primary education and categorized as low (<6 yrs), middle (6–10 yrs), and high (>10 yrs) [9,23]. 

### 2.6. Assessment of Potential Confounders

Potential covariates that might influence the association between dietary patterns and BMI, underweight, and overweight/obesity at age 10 were the children’s exact age at the 5-year height and weight measurement, sex, maternal BMI, screen time, and ethnicity. Maternal BMI and screen time were collected via the ‘5-year questionnaire’. Maternal BMI (kg/m^2^) was based on mothers’ self-reported height and weight when the child was 5 years old. Screen time (h/day) was based on the duration in hours that the child spent watching TV or using a computer or console per day at age 5 [24]. Ethnicity was defined using data from the ‘pregnancy questionnaire’ and was based on the country of birth of the pregnant woman and her mother, including both first-generation women (born outside the Netherlands) and second-generation women (born in the Netherlands but whose mother was born in another country). We defined five groups: Dutch, Turkish, African Surinamese, Moroccan, and “other” ethnicities (mostly of non-Western origin). 

### 2.7. Statistical Analysis 

Population and anthropometric characteristics were described in numbers with percentages or means with standard deviations (SDs) for each maternal education level separately. ANOVA and post hoc Bonferroni were applied to show differences by maternal education group.

For the second research objective, linear regression was used to determine to which extent the RRR-derived dietary patterns at age five were associated with the BMI z-scores at age 10. Logistic regression was used to determine to which extent the RRR-derived dietary patterns were related to underweight and overweight/obesity at age 10. Of each dietary pattern, the individual pattern score was used as the continuous independent variable and the BMI z-score at age 10, underweight at age 10, and overweight/obesity at age 10 were used as the dependent variables (Model 1; crude). In the adjusted model (Model 2), the association was adjusted for children’s exact age at the 5-year height and weight measurement, sex, maternal BMI at age 5, screen time at age 5, and ethnicity. The RRR analyses were performed in SAS^®^ OnDemand for Academics version STAT 15.2. The other analyses were performed in IBM SPSS Statistics version 28 for Windows. The level of statistical significance was 0.05. 

## 3. Results

### 3.1. Population Characteristics 

Table 1 presents the characteristics of the study population (n ≤ 1728) stratified by the level of maternal education. Most children (68.3%) had higher-educated mothers, 19.8% middle and 11.8% had mothers with a lower education level. Mean (SD) age was 5.9 (0.5) in the low, 5.7 (0.5) in the middle, and 5.6 (0.4) years in the children of higher-educated mothers at the 5-year health check. The highest BMI z-scores at age 5 were observed in the group of lower (0.42 SD 1.21) and middle-educated mothers (0.08 SD 1.04) and the lowest BMI z-scores were observed in the group of higher-educated mothers (−0.06 SD 0.81).

### 3.2. RRR-Derived Dietary Patterns by Maternal Education 

In each group, the first dietary pattern was positively associated with the BMI z-score and FMI. In the children with higher-educated mothers, pattern 1 was also positively correlated with FFMI. The explained variances in response variables were 15.4%, 12.2%, and 4.9% for the low, middle, and high maternal education groups, respectively. We observed differences as well as similarities in food groups characterizing the RRR-derived dietary patterns between each group (Table 2). Food groups that are typical for children of lower-educated mothers were water/tea with sugar, savory snacks, low-fat meat and fruits (positive loadings), and sweet sandwich toppings, tomato sauce, peanut butter, whole-grain warm meals, healthy snacks, and high-fat meat (negative loadings). Water/tea, low-fat cheese, fish, low-fat dairy, fruit drinks, eggs and low-fat meat (positive loadings), and boiled potatoes and healthy snacks (negative loading) were typical for children of middle–high-educated mothers. For the children of higher-educated mothers, typical food groups included low-fat cheese, fruits, whole-grain breakfast products, low-fat and processed meat (positive loadings), and full-fat dairy and tomato sauce (negative loading). Water/tea, low-fat meat, low-fat cheese and fruits (positive loadings), and tomato sauce for pasta and healthy snacks (negative loadings) were representative of RRR-derived pattern 1 in multiple groups. For comparison reasons, Supplemental Table S1 provides an overview of all the factor loadings on the RRR-derived dietary pattern 1 in the total ABCD cohort (n ≤ 1728) as well as in each level of maternal education.

The second dietary pattern was positively associated with FFMI in all the groups, and negatively associated with FMI in the higher maternal education group. The explained variances in the response variables for the second pattern were 8.09% (low), 3.70% (middle), and 1.57% (higher maternal education level). The food groups characterizing RRR-derived pattern 2, stratified by the level of maternal education, are shown in Supplemental Table S2. Additionally, Supplemental Table S3 provides an overview of all the factor loadings on the RRR-derived dietary pattern 2 in the total ABCD cohort (n ≤ 1728) and in each group separately.

### 3.3. RRR-Derived Dietary Patterns Related to Bmi Z-Scores and Weight Status at Age 10 

In the children of lower- and middle-educated mothers, the mean BMI z-score at age 10 was higher (M ≤ 0.64 [SD ≤ 1.29] and M ≤ 0.23 [1.16], respectively) compared to age 5, while in the children of higher-educated mothers, the mean BMI z-score was lower at age 10 (M = −0.17 [0.96]). 

In all the groups, the percentage of children with overweight or obesity at age 10 was higher compared to age 5, although the percentage was highest in the children of lower-educated mothers (34.8% vs. 16.9% in middle and 6.4% in high at age 10 and 25% vs. 11.5% in middle and 5.1% in children of higher-educated mothers at age 5). 

In the children of lower and middle-educated mothers, the percentage of underweight children at age 10 was lower than at age 5 (13.2% and 15.1%, respectively, at age 5 and 10.3% and 11.1%, respectively, at age 10) while the percentages of underweight children in higher-educated mothers were 12.9% at both age 5 and 10. 

In all the groups, pattern 1 was associated with higher BMI z-scores at age 10 and higher odds of being overweight or obese at age 10 with the strongest associations observed in the children of lower-educated mothers (β ≤ 0.43 [95% CI ≤ 0.21; 0.66], *p* ≤ 0.001 for BMI z-score and OR overweight/obesity ≤ 2.21 [1.32; 3.72], *p* ≤ 0.003), and with a lower odds of being underweight (Table 3). However, these associations were only statistically significant in children of higher-educated mothers (OR underweight ≤ 0.66 [0.53; 0.81], *p* ≤ 0.001). Pattern 2 was mainly associated with lower odds of being overweight/obese at age 10 (OR ≤ 0.66 [0.52; 0.83], *p* ≤ 0.001) in children of higher-educated mothers only (Supplemental Table S4). 

## 4. Discussion

In a large population-based cohort study in young children, we used RRR to derive dietary patterns stratified by the level of maternal education that explain the variation in BMI z-score, FMI, and FFMI at age 5. We observed similarities as well as differences in the food groups that characterized each dietary pattern. Yet, RRR-derived pattern 1 was positively associated with BMI z-scores and higher odds of being overweight or obese at age 10 in all the groups. 

### 4.1. Interpretation and Comparison with Previous Studies 

In each group, we identified a dietary pattern that was correlated with BMI and FMI and a dietary pattern that was correlated with FFMI. To the best of our knowledge, we did not find other studies deriving dietary patterns in children stratified by the level of maternal education. Generally, dietary patterns are derived within the total population and subsequently associated with (measures of) SES [5,7,8,9]. As differences in BMI are unequally distributed across SES groups [1,2], the derived dietary patterns could potentially give insight into which food groups contribute to the variance in BMI, FMI, and FFMI in young children from mothers with different education levels. Indeed, our analysis indicates that although there were some similarities, different food groups contributed to the dietary pattern associated with increased body weight at the follow-up (age 10). In our study, the consumed food items were reduced to 41 food groups based on culinary use and nutritional value [9]. Each food item in the Food Frequency Questionnaire was linked with at least one food item in the Dutch Food Composition Database [20]. It might be possible that food items are consumed in different combinations implicating possible different classifications of food groups than used in our study. For example, the food group ‘sugar’ consist of sugar consumed as a sweetener in yogurt or cereals, but also as a sweetener consumed in tea. In the children of lower-educated mothers, both the intake of water/tea (0.25) and sugar (0.22) were of importance for pattern 1. In the middle group, however, the intake of water/tea was of importance (0.35) while the intake of sugar (−0.10) did not characterize pattern 1. Sensitivity analyses show that in the children of lower-educated mothers, more children consumed sugar as a sweetener in tea (75%) compared to 67% in the middle- and 40% in the children of higher-educated mothers, and that the average intake was highest in consumers in the lower (2.55 (2.70) compared to the middle (2.18 (2.60)) and higher maternal education groups (1.47 (2.12)). These observations possibly reflect the different culinary uses of the food items between the different groups. In the children of lower-educated mothers, the possible use of tea and sugar in tea as a sweetener could be combined in a food group ‘sugar-sweetened beverages’. However, interestingly, we observed in all the groups that pattern 1 was mainly characterized by a high intake of food items generally considered as healthy which could not be further explained by the possible differences in culinary use between the groups. In the lower maternal education group, these items were water/tea, savory snacks, sugar, low-fat meat, and fruits. In the middle group, these items were water/tea, low-fat cheese, fish, low-fat dairy, fruit drinks, eggs, and low-fat meat and in the higher maternal education group, these items were low-fat cheese, fruits, whole-grain breakfast products, low-fat meat, and processed meat (Table 2). The Dutch Generation R cohort has also derived a dietary pattern associated with a higher FFMI (high intakes of whole-grain products, pasta, rice, fruit, dairy, vegetable oils and fats, and non-sugar-containing beverages) and a dietary pattern that was associated with both FMI and FFMI (high intakes of refined grain products, potatoes, meat, fish, soups, sauces, and sugar-containing beverages) [16]. However, these patterns were derived at age 1 and unlike our results, the FMI pattern was not characterized with food items generally considered as healthy. It might be possible that parents provide their children with more ‘healthy food items’ when children are already overweight, which possibly indicates reverse causality. From birth, the weight development of children is monitored through Youth Health Care (YHC). Possibly, the YHC gave nutritional advice if the weight development of children was suboptimal, which could have resulted in adjusted nutritional habits. Sensitivity analyses showed that in all the groups, all the food items that were positively related to pattern 1 were consumed in higher amounts by the children with higher BMI z-scores at age 5 (BMI z-scores > 1) than by the children with lower BMI z-scores (BMI z-scores < 1). Food items that were negatively related to pattern 1 were consumed in lower amounts by the children with higher BMI z-scores. Food items that were positively related to pattern 2 did not show a clear pattern. In the total population and in children of higher-educated mothers, ‘traditional healthy bread items’, e.g., whole-grain breakfast products and full-fat spreads, were related to the variation in FFMI.

The longitudinal results showed that in each group, a positive association was found between the RRR-derived pattern 1 and BMI z-scores and the risk of overweight or obesity at age 10, which was strongest in the lower maternal education group, while only in the higher maternal education group, the RRR-derived pattern 2 was associated with lower odds of being overweight/obese at age 10. We observed increasing associations with decreasing levels of maternal education (pattern 1: low (β ≤ 0.43 [95% CI ≤ 0.21; 0.66] and high (β ≤ 0.24 [95% CI ≤ 0.18; 0.30] and pattern 2: low (β ≤ 0.22 [95% CI = −0.01; 0.46] and high (β = −0.05 [95% CI = −0.10; −0.005]) which is in line with, and could be partly explained by the observed increasing percentages of the explained variances of the response variables in the lower maternal education group. Our results underline that the derived food patterns at age 5 are strongly associated with the risk of overweight development at age 10 in children of lower-educated mothers than children in other groups. The observed longitudinal results are in line with earlier results in our cohort that showed that higher scores on a PCA-derived ‘healthy’ pattern at age 5 (also associated with high intakes of water/tea, fruits, and fish and low intakes of sweet sandwich toppings) were also associated with significantly higher BMI z-score at age 10 in all the SES groups [13]. Higher scores on a ‘full-fat’ pattern at age 5 (also high intakes of full-fat spreads) were associated with significantly lower BMI at age 10 in high SES children [13]. These findings are also in line with results from the Generation R cohort, where the FFMI pattern was positively associated with FFMI at age 6 but not with FMI [16] and these associations remained up to age 10 [25]. A higher diet quality at the age of 1 and 8 years old was (independent of diet quality of the other time point) associated with higher BMI until the age of 10 years and was explained by a higher FFMI [25]. Unlike few studies [16,25,26] but in line with another study [27] we did not observe indications that the association between ‘healthy dietary patterns’ and BMI in children would be explained by the amount of FFMI rather than FMI. 

In our cohort, interestingly, only in children of higher-educated mothers, pattern 2 was associated with lower BMI z-scores at age 10 and lower FMI at age 12 (Supplemental Tables S5 and S6). These results suggest that in this group of children, possibly other protecting mechanisms are present that prevent the development of overweight at age 10. The results in our cohort also show that in the children of higher-educated mothers, the mean BMI z-score at age 10 was lower than in the children of lower and middle high educated mothers, which is in line with general observations [28,29].

### 4.2. Methodological Considerations 

A strength of this study is the population-based cohort-design that included a number of 1728 children with complete data on diet, maternal education, BMI, FMI, and FFMI at age 5 and BMI at age 10 years. In other studies using RRR, generally, nutrients [17] or disease-specific intermediates [30] were used as response variables. As childhood obesity is unequally distributed across socio-economic status (SES) groups, we target this public health problem by using BMI as well as FMI and FFMI as the response variables. Body mass includes fat mass, lean mass, and bone mass [31,32]. Therefore, increased BMI could be caused by increased fat mass or increased lean mass, or a combination [33]. Analyses stratified per level of maternal education, used as a proxy for SES, gave an overview of dietary patterns that explain the differences in the variation in BMI and other measures of body composition in each group. 

Height, weight, and components of body composition were measured by trained researchers and health professionals using standard protocols. Data on BMI were available when children were 5, 10, and 12 years old. FMI and FFMI were not available at age 10, but were available at age 5 and in a select population at age 12. Longitudinal analyses describing the observed associations between the dietary patterns and BMI, FMI, and FFMI in each group were conducted as sensitivity analyses. We observed comparable associations at age 10 and age 12. In each group, pattern 1 was positively associated with FMI at age 12 while pattern 2 was negatively associated with FMI at age 12 in the children of higher-educated mothers. The results are shown in Supplemental Tables S5 and S6.

There are also a few possible limitations. Possibly, some biases may have been introduced into the analyses, especially because the non-responders came more often from lower maternal education groups. Response rates per SES group were 11% for the low plus middle SES group combined and 38% for the high SES group. A non-response analysis determining the level of selective response and selection bias between pregnancy and birth outcomes indicated that selective non-response was present in the ABCD cohort, but that the observed selection bias was acceptably low and did not influence the studied birth outcomes [34]. Also in our cohort, children from lower-educated mothers came disproportionally from non-native ethnicities (Table 1). Despite the fact that ethnicity and SES are correlated, we observed in previous research that the effect of maternal education level is not only driven by ethnicity [9,13]. We observed that both ethnicity and maternal education explained differences in dietary pattern scores between groups at age 5 y which may suggest that both are relevant for the adherence to a specific dietary pattern [9]. Previous results also showed different prevalences of overweight and obesity within the non-native group despite comparable SES levels [13]. For example, children of Turkish origin (87% low-/middle-educated mothers) developed more often (44%) overweight and obesity between the ages of 5 and 10 years than children of Moroccan origin (24%; 82% low-/middle-educated mothers). 

Dietary intake was obtained using an FFQ, which may be subject to measurement errors. Mothers were asked to report all that their child had eaten or drunk in the last four weeks. In the Netherlands, parents provide their children with all the consumed foods and drinks while attending primary school. If children were looked after by a childminder or went to after-school care, mothers were asked to ask the person responsible what their child ate or drank there. Nevertheless, mothers may not be fully aware of their children’s exact consumption at all times during the last 4 weeks. The FFQ that we used showed reasonable to good validity for estimating energy intake in a previous validation study against the doubly labeled water method [19]. Longitudinal analyses were adjusted for important confounding factors such as children’s exact age at the 5-year height and weight measurement, sex, maternal BMI, screen time, and ethnicity. However, in addition, there may be more potential confounding factors (e.g., maternal age, health-conscious behavior of the mother during pregnancy, parity, breastfeeding, and complementary feeding) that are possibly associated with early life nutrition [35,36]. 

## 5. Conclusions

Our results indicate that the observed dietary patterns stratified by the level of maternal education contribute to the variation in BMI, FMI, and FFMI at age 5 and are relevant for longitudinal BMI and the odds of being overweight or obese at age 10. However, our results also indicate that the possibility of reversed causality could not be ruled out. To investigate whether parents possibly provide their children with more ‘healthy food items’ when children are already overweight, it is relevant to have nutritional, weight, and body composition data below the age of 5 years old. In further analyses, also the use of additional potential confounding factors could be investigated to study the association between dietary patterns and BMI and body composition in younger children. We observed that each dietary pattern was characterized by both similar and different food groups. However, these patterns were also characterized by only a few food groups and one may argue if these food groups actually represent a dietary pattern. Despite the observed results in our study, the observed dietary patterns do not necessarily give insights for practical implementations. 

## Figures and Tables

**Table 1:** Population characteristics of the ABCD study population at age 5 and 10 years by maternal education level (n ≤ 1728).

		Maternal Education Level ^a^		
		Low (n ≤ 204)	Middle (n ≤ 343)	High (n ≤ 1181)
Age, in years, mean (SD)	Age 5	5.9 (0.5) *	5.7 (0.5) *	5.6 (0.4) *
	Age 10	10.6 (0.4)	10.7 (0.4)	10.6 (0.4)
Sex, n (%) boys		108 (52.9)	178 (51.9)	574 (48.6)
Screen time, in h/day ^b^, mean (SD)	Age 5	2.30 (1.21) *	1.79 (1.04) *	1.17 (0.75) *
Maternal BMI, in kg/m^2 c^, mean (SD)		26.6 (5.7) *	24.9 (5.0) *	22.9 (3.3) *
Ethnicity, n (%)				
Dutch		83 (40.7) *	235 (68.5) *	1053 (89.2) *
African Surinamese		18 (8.8) *	34 (9.9) *	24 (2.0) *
Turkish		22 (10.8) *	16 (4.7) *	6 (0.5) *
Moroccan		44 (21.6) *	32 (9.3) *	17 (1.4) *
Other ethnicities		37 (18.1) *	26 (7.6) *	81 (6.9) *
BMI z-score, mean (SD)	Age 5	0.42 (1.21) *	0.08 (1.04)	−0.06 (0.81)
	Age 10	0.64 (1.29) *	0.23 (1.16) *	−0.17 (0.96) *
FFMI, in kg/m^2^, mean (SD)	Age 5	12.19 (0.84)	12.25 (0.83)	12.26 (0.80)
FMI, in kg/m^2^, mean (SD)	Age 5	3.97 (1.84) *	3.32 (1.43) *	3.01 (1.03) *

^a^ level of maternal education was defined as the number of years of post-primary education: low (<6 yrs), middle (6–10 yrs), and high (>10 yrs); ^b^ data on screen time was missing for n ≤ 7 subjects; ^c^ data on maternal BMI was missing for n ≤ 88 subjects. * significant (*p* < 0.05) with all the groups.

**Table 2:** Food groups with factor loadings > 0.20 on the RRR-derived dietary pattern 1 with the BMI z-score, FMI, and FFMI at age 5 as response variables in the ABCD cohort (n ≤ 1728).

Maternal Education Level					
Low (n ≤ 204)		Middle (n ≤ 343)		High (n ≤ 1181)	
Food Group	Load	Food Group	Load	Food Group	Load
Positive loadings					
Water/tea	0.25	Water/tea	0.35	Low-fat cheese	0.37
Savory snacks	0.24	Low-fat cheese	0.32	Fruits	0.33
Sugar	0.22	Fish	0.28	Whole grain breakfast products	0.22
Low-fat meat	0.20	Low-fat dairy	0.28	Low-fat meat	0.21
Fruits	0.20	Fruit drink	0.25	Processed meats	0.21
		Eggs	0.24		
		Low-fat meat	0.24		
Negative loadings					
Sandwich toppings sweet	−0.33	Boiled potatoes	−0.22	Full-fat dairy	−0.40
Tomato sauce for pasta	−0.27	Healthy snacks	−0.21	Tomato sauce for pasta	−0.23
Peanut butter	−0.23				
Whole-grain warm meals	−0.21				
Healthy snacks	−0.21				
High-fat meat	−0.20				
Variance explained in food intake, %	2.64		2.79		3.06
Variance explained in response variables, %	15.39		12.21		4.90

**Table 3:** The association between the RRR-derived dietary pattern 1 at age 5 and BMI z-score and weight status at age 10 by the level of maternal education (n ≤ 1728).

	BMI Z-Score		Underweight		Overweight/Obesity	
Level of Maternal Education:	B (95%-CI) ^a^	*p*-Value	OR (95%-CI)	*p*-Value	OR (95%-CI)	*p*-Value
Low (n ≤ 204)						
Model 1: crude	0.50 (0.28; 0.72)	<0.001	0.45 (0.25; 0.81)	0.008	2.57 (1.65; 4.00)	<0.001
Model 2: adjusted ^b^	0.43 (0.21; 0.66)	<0.001	0.52 (0.26; 1.03)	0.06	2.21 (1.32; 3.72)	0.003
Middle (n ≤ 343)						
Model 1: crude	0.40 (0.27; 0.53)	<0.001	0.60 (0.39; 0.93)	0.02	2.06 (1.50; 2.83)	<0.001
Model 2: adjusted ^b^	0.23 (0.09; 0.36)	0.001	0.73 (0.46; 1.16)	0.18	1.52 (1.06; 2.19)	0.02
High (n ≤ 1181)						
Model 1: crude	0.26 (0.20; 0.32)	<0.001	0.65 (0.53; 0.78)	<0.001	1.76 (1.37; 2.25)	<0.001
Model 2: adjusted ^b^	0.24 (0.18; 0.30)	<0.001	0.66 (0.53; 0.81)	<0.001	1.81 (1.37; 2.38)	<0.001

^a^ regression coefficients reflect a change in the BMI z-score at age 10 for a 1-unit increase in dietary pattern score; ^b^ model 2 is adjusted for children’s exact age at the 5-year height and weight measurement, sex, maternal BMI, screen time at age 5, and ethnicity; because of missing values, n ≤ 1635 subjects (low n ≤ 173, middle n ≤ 326, and high n ≤ 1136) were included in the adjusted analyses.

## Data Availability

The datasets generated and/or analyzed during the current study are not publicly available due to ethical restrictions related to protecting patient confidentially, but are available from the corresponding author upon reasonable request.

## Supplementary Materials

The following supporting information can be downloaded at: https://www.mdpi.com/article/10.3390/nu16193242/s1, Table S1. Overview of factor loadings on the RRR-derived dietary pattern 1 with BMI z-score, FMI, and FFMI at age 5 as response variables in the ABCD cohort (n ≤ 1728); Table S2. Overview of food groups with factor loadings >0.20 on the RRR-derived dietary pattern 2 with BMI z-score, FMI, and FFMI at age 5 as response variables in the ABCD cohort (n ≤ 1728); Table S3. Factor loadings on the RRR-derived dietary pattern 2 with BMI z-score, FMI, and FFMI at age 5 as response variables in the ABCD cohort (n ≤ 1728); Table S4. The association between RRR-derived dietary pattern 2 at age 5 and BMI z-score and weight status at age 10 by level of maternal education (n ≤ 1728); Table S5. The association between RRR-derived dietary pattern 1 at age 5 and BMI z-score, body composition, and weight status at age 12 by the level of maternal education (n ≤ 525); Table S6. The association between RRR-derived dietary pattern 2 at age 5 and BMI z-score, body composition, and weight status at age 12 by level of maternal education (n ≤ 525).
