# Supplementary material for: Dietary Patterns by Level of Maternal Education and Their Contribution to BMI, Fat Mass Index, and Fat-Free Mass Index at Age 5 and the Longitudinal Association with BMI at Age 10
Source: Nutrients. 2024 Sep 25;16(19):3242. doi: 10.3390/nu16193242 (PMC11479119; doi:10.3390/nu16193242)
Supplement: Supplementary file 1 [file nutrients-16-03242-s001.zip › nutrients-3166228-supplementary.pdf]

Supplemental Table S1. Overview of factor loadings on the RRR-derived dietary pattern 1 with BMI z-score, FMI and FFMI at age 5 as response variables in the ABCD cohort (n=1728).

		Level of maternal education		
**Food group**	**Total cohort (n=1728)**	**Low (n=204)**	**Middle (n=343)**	**High (n=1181)**
Fruits	0.14	0.20	0.03	0.33
Vegetables	0.01	0.07	0.13	0.15
Refined breakfast products	0.11	-0.13	0.01	0.00
Whole grain breakfast products	-0.07	0.17	0.03	0.22
Low-fat spreads	0.00	0.06	-0.16	0.17
Full-fat spreads	-0.15	-0.15	-0.02	-0.07
Low-fat cheese	0.32	0.18	0.32	0.37
Full-fat cheese	0.04	-0.13	0.02	0.14
Processed meats	0.02	0.10	-0.13	0.21
Peanut butter	-0.23	-0.23	-0.11	-0.11
Sandwich toppings (sweet)	-0.32	-0.33	-0.17	-0.13
Low-fat dairy	0.14	0.05	0.28	0.16
Medium-fat dairy	-0.01	0.02	-0.09	0.09
Full-fat dairy	-0.17	-0.17	-0.08	-0.40
Unhealthy meals	0.04	0.07	-0.08	0.05
Healthy meals	0.07	-0.03	-0.03	0.16
Tomato sauce (for pasta)	-0.23	-0.27	0.02	-0.23
Refined grain products	-0.02	-0.19	0.14	-0.17
Whole grain products warm meal	-0.12	-0.21	-0.15	0.04
Fried potato products	0.02	-0.06	-0.09	-0.16
Boiled potatoes	-0.02	0.09	-0.22	-0.08
Sauces	0.25	0.08	0.13	0.05
Pulses	0.14	0.11	0.17.	-0.05
Fish	0.23	0.09	0.28	0.16
Eggs	0.21	0.16	0.24	0.05
Low-fat meat	0.29	0.20	0.24	0.21
High-fat meat	-0.13	-0.20	-0.07	-0.09
Meat alternatives and soy products	-0.03	0.00	-0.05	0.08
Granola bars	0.01	0.19	0.03	-0.06
Biscuits and pastries	0.12	-0.03	0.12	0.06
Ice cream	-0.01	0.02	-0.09	-0.11
Chocolate and candy	0.04	0.05	-0.03	-0.10
Healthy snacks	-0.09	-0.21	-0.21	0.06
Savory snacks	0.27	0.24	0.09	-0.05
Nuts	0.06	0.08	0.12	-0.04
Sugar sweetened sodas	0.06	0.17	-0.06	-0.07
Artificially sweetened beverages	0.12	0.05	0.16	0.09
Fruit drink	0.18	0.18	0.25	-0.17
Fruit drink concentrate	-0.19	0.04	-0.18	-0.02
Water and tea	0.30	0.25	0.35	0.16
Sugar	0.04	0.22	-0.10	-0.13

Supplemental Table S2. Overview of food groups with factor loadings > 0.20 on the RRR derived dietary pattern 2 with BMI z-score, FMI and FFMI at age 5 as response variables in the ABCD cohort (n=1728).

**Level of maternal education**					
**Low (n=204)**		**Middle (n=343)**		**High (n=1181)**	
**Food group**	**Load**	**Food group**	**Load**	**Food group**	**Load**
Positive loadings					
Tomato sauce for pasta	0.32	High-fat meat	0.31	Granola bars	0.28
Fried potato products	0.33	Vegetables	0.27	Whole grain breakfast products	0.27
		Fruit drink concentrate	0.23	Sandwich toppings sweet	0.25
		Granola bars	0.21	Full-fat spreads	0.20
		Medium-fat dairy	0.21		
		Sauces	0.20		
Negative loadings					
Eggs	-0.34	Tomato sauce for pasta	-0.34	Savory snacks	-0.35
Low-fat meat	-0.33	Biscuits/pastries	-0.21	Biscuits/pastries	-0.31
Full-fat dairy	-0.23	Low-fat meat	-0.20	Sauces	-0.28
Pulses	-0.23	Unhealthy meals	-0.20	Sugar sweetened sodas	-0.20
				Sugar	-0.20
				Artificially sweetened beverages	-0.20
Variance explained in food intake, %	2.35		2.43		3.79
Variance explained in response variables, %	8.09		3.70		1.57

Supplemental Table S3. Factor loadings on the RRR-derived dietary pattern 2 with BMI z-score, FMI and FFMI at age 5 as response variables in the ABCD cohort (n=1728).

	Level of maternal education			
**Food group**	**Total cohort (n=1728)**	**Low (n=204)**	**Middle (n=343)**	**High (n=1181)**
Fruits	0.25	0.12	0.09	0.11
Vegetables	0.21	0.16	0.27	0.03
Refined breakfast products	-0.06	-0.04	0.07	-0.09
Whole grain breakfast products	0.30	0.18	-0.08	0.27
Low-fat spreads	0.07	-0.15	0.07	0.03
Full-fat spreads	0.10	-0.02	-0.09	0.20
Low-fat cheese	0.15	0.14	-0.17	0.00
Full-fat cheese	0.05	-0.07	0.13	-0.02
Processed meats	0.19	-0.10	0.11	0.15
Peanut butter	0.05	-0.12	-0.08	0.17
Sandwich toppings (sweet)	0.09	-0.08	-0.09	0.25
Low-fat dairy	0.08	-0.05	0.05	0.01
Medium-fat dairy	0.17	0.17	0.21	0.06
Full-fat dairy	-0.32	-0.23	-0.18	-0.06
Unhealthy meals	-0.03	0.07	-0.20	-0.02
Healthy meals	0.07	0.17	0.13	-0.11
Tomato sauce (for pasta)	-0.15	0.32	-0.34	-0.12
Refined grain products	-0.21	-0.14	-0.06	-0.17
Whole grain products warm meal	0.00	0.04	-0.16	0.01
Fried potato products	-0.10	0.33	-0.15	-0.16
Boiled potatoes	-0.11	0.11	-0.04	-0.12
Sauces	-0.11	-0.02	0.20	-0.28
Pulses	0.00	-0.23	0.18	0.03
Fish	-0.06	-0.08	0.03	-0.18
Eggs	-0.18	-0.34	-0.02	-0.16
Low-fat meat	-0.08	-0.33	-0.20	0.03
High-fat meat	0.10	0.11	0.31	0.00
Meat alternatives and soy products	0.06	0.10	-0.13	0.05
Granola bars	0.23	0.11	0.21	0.28
Biscuits and pastries	-0.23	-0.01	-0.21	-0.31
Ice cream	-0.13	0.11	-0.08	-0.17
Chocolate and candy	-0.18	0.07	-0.15	-0.17
Healthy snacks	0.14	0.17	0.18	0.03
Savory snacks	-0.24	-0.17	0.19	-0.35
Nuts	-0.11	-0.02	-0.12	-0.09
Sugar sweetened sodas	-0.22	-0.14	0.00	-0.20
Artificially sweetened beverages	-0.10	-0.07	-0.01	-0.20
Fruit drink	-0.06	-0.02	0.08	0.01
Fruit drink concentrate	0.14	0.16	0.23	0.05
Water and tea	-0.11	-0.13	-0.12	-0.14
Sugar	-0.28	-0.17	-0.13	-0.20

Supplemental Table S4. The association between RRR-derived dietary pattern 2 at age 5 and BMI z-score and weight status at age 10 by level of maternal education (n=1728).

	BMI z-score		Underweight		Overweight/obesity	
Level of maternal education	**B (95%-CI)[a]**	**p-value**	**OR (95%-CI)**	**p-value**	**OR (95%-CI)**	**p-value**
**Low (n=204)**						
Model 1: crude	0.09 (-0.14; 0.33)	0.44	0.65 (0.35; 1.21)	0.17	1.08 (0.74; 1.59)	0.69
Model 2: adjusted[b]	0.22 (-0.01; 0.46)	0.07	0.40 (0.17; 0.93)	0.03	1.16 (0.74; 1.82)	0.52
**Middle (n=343)**						
Model 1: crude	0.13 (-0.01; 0.27)	0.07	0.61 (0.41; 0.90)	0.01	1.13 (0.82; 1.57)	0.45
Model 2: adjusted[b]	0.11 (-0.03; 0.24)	0.13	0.60 (0.38; 0.96)	0.03	1.08 (0.75; 1.54)	0.69
**High (n=1181)**						
Model 1: crude	-0.06 (-0.11; -0.002)	0.04	1.02 (0.87; 1.20)	0.81	0.64 (0.52; 0.78)	<0.001
Model 2: adjusted[b]	-0.05 (-0.10; 0.005)	0.08	1.03 (0.85; 1.24)	0.77	0.66 (0.52; 0.83)	<0.001

[a] Regression coefficients reflect a 1-unit change in BMI z-score at age 10 for each increment in dietary pattern score; [b] Model 2 is adjusted for sex, children's exact age at the 5-years height and weight measurement, screen time at age 5, maternal BMI at age 5 and ethnicity, because of missing values n=1635 subjects (low n=173, middle n=326, high maternal education level n=1136) were included in the adjusted analyses.

Supplemental Table S5. The association between RRR-derived dietary pattern 1 at age 5 and BMI z-score, body composition and weight status at age 12 by level of maternal education (n=525).

	BMI z-score	FMI	FFMI	Underweight	Overweight
Level of maternal education	B (95%-CI)[a]	B (95%-CI)[a]	B (95%-CI)[a]	OR (95%-CI)	OR (95%-CI)
**Low (n=48)**					
Model 1: crude	0.31 (-0.15; 0.77)	0.80 (0.01; 1.58)	0.27 (-0.48; 1.02)	2.85 (0.56; 14.5)	2.21 (0.97; 5.00)
Model 2: adjusted[b]	0.20 (-0.24; 0.65)	0.78 (0.01; 1.55)	0.07 (-0.65; 0.79)	8.85 (0.06; inf)	2.70 (0.87; 8.38)
**Middle (n=106)**					
Model 1: crude	0.37 (0.13; 0.61)	0.79 (0.35; 1.23)	0.24 (-0.09; 0.57)	0.52 (0.19; 1.45)	2.25 (1.28; 3.95)
Model 2: adjusted[b]	0.18 (-0.08; 0.43)	0.52 (0.06; 0.99)	0.03 (-0.31; 0.37)	1.14 (0.29; 4.50)	1.76 (0.82; 3.75)
**High (n=370)**					
Model 1: crude	0.20 (0.08; 0.31)	0.16 (0.02; 0.30)	0.16 (0.01; 0.31)	0.64 (0.47; 0.89)	1.08 (0.60; 1.94)
Model 2: adjusted[b]	0.17 (0.05; 0.28)	0.21 (0.07; 0.34)	0.08 (-0.07; 0.22)	0.64 (0.46; 0.89)	1.06 (0.58; 1.92)

[a] Regression coefficients reflect a 1-unit change in BMI z-score, FMI and FFMI at age 12 for each increment in dietary pattern score; [b] Data on FMI and FFMI was missing for n=7 subjects (n=1 middle SES, n=6 high SES) [c] Model 2 is adjusted for sex, children's exact age at the 5-years height and weight measurement, screen time at age 5, maternal BMI at age 5 and ethnicity, because of missing values n=505 subjects (low n=45, middle n=105, high maternal education level n=355) were included in the adjusted analyses.

Supplemental Table S6. The association between RRR-derived dietary pattern 2 at age 5 and BMI z-score, body composition and weight status at age 12 by level of maternal education (n=525).

	**BMI z-score**	**FMI[b]**	**FFMI[b]**	**Underweight**	**Overweight**
Level of maternal education	**B (95%-CI)[a]**	**B (95%-CI)[a]**	**B (95%-CI)[a]**	**OR (95%-CI)**	**OR (95%-CI)**
**Low (n=48)**					
Model 1: crude	0.06 (-0.39; 0.51)	-0.27 (-1.05; 0.51)	0.44 (-0.26; 1.15)	1.08 (0.26; 4.44)	1.33 (0.65; 2.72)
Model 2: adjusted[c]	0.06 (-0.39; 0.50)	-0.22 (-1.03; 0.60)	0.34 (-0.37; 1.05)	11.4 (0.12; inf)	1.67 (0.62; 4.48)
**Middle (n=106)**					
Model 1: crude	-0.11 (-0.37; 0.16)	-0.18 (-0.67; 0.31)	0.06 (-0.29; 0.41)	0.94 (0.40; 2.19)	1.28 (0.70; 2.37)
Model 2: adjusted[c]	-0.01 (-0.64; 0.24)	0.10 (-0.37; 0.57)	0.05 (-0.28; 0.39)	0.53 (0.12; 2.36)	1.56 (0.73; 3.34)
**High (n=370)**					
Model 1: crude	-0.04 (-0.14; 0.05)	-0.20 (-0.32; -0.08)	0.08 (-0.05; 0.21)	1.05 (0.82; 1.34)	0.89 (0.55; 1.44)
Model 2: adjusted[c]	-0.04 (-0.14; 0.06)	-0.14 (-0.26; -0.03)	0.06 (-0.06; 0.18)	1.07 (0.82; 1.40)	0.85 (0.50; 1.47)

[a] Regression coefficients reflect a 1-unit change in BMI z-score, FMI and FFMI at age 12 for each increment in dietary pattern score; [b] Data on FMI and FFMI was missing for n=7 subjects (n=1 middle SES, n=6 high SES) [c] Model 2 is adjusted for sex, children's exact age at the 5-years height and weight measurement, screen time at age 5, maternal BMI at age 5 and ethnicity, because of missing values n=505 subjects (low n=45, middle n=105, high level of maternal education n=355) were included in the adjusted analyses.
